# Tailoring recommendation algorithms to ideal preferences makes users better off

**DOI:** 10.1038/s41598-023-34192-x

**Published:** 2023-06-08

**Authors:** Poruz Khambatta, Shwetha Mariadassou, Joshua Morris, S. Christian Wheeler

**Affiliations:** 1 Anderson School of Management, University of California, Los Angeles, Los Angeles, CA 90095 USA; 2 Graduate School of Business, Stanford University, Stanford, CA 94305 USA

**Keywords:** Psychology, Human behaviour

## Abstract

People often struggle to do what they ideally want because of a conflict between their actual and ideal preferences. ​​​By focusing on maximizing engagement, recommendation algorithms appear to be exacerbating this struggle. However, this need not be the case. Here we show that tailoring recommendation algorithms to ideal (vs. actual) preferences would provide meaningful benefits to both users and companies. To examine this, we built algorithmic recommendation systems that generated real-time, personalized recommendations tailored to either a person’s actual or ideal preferences. Then, in a high-powered, pre-registered experiment (*n* = 6488), we measured the effects of these recommendation algorithms. We found that targeting ideal rather than actual preferences resulted in somewhat fewer clicks, but it also increased the extent to which people felt better off and that their time was well spent. Moreover, of note to companies, targeting ideal preferences increased users' willingness to pay for the service, the extent to which they felt the company had their best interest at heart, and their likelihood of using the service again. Our results suggest that users and companies would be better off if recommendation algorithms learned what each person was striving for and nudged individuals toward their own unique ideals.

## Introduction

Social media algorithms recommend content to billions of people every day. These recommendations are tailored to user preferences, yet it has become increasingly clear that these algorithms can have harmful effects on individuals and society^1–3^. This may in part be due to the types of preferences that these algorithms target.

On topics as varied as social groups, politicians, policy issues, companies, behaviors, and relationship partners, people commonly hold an actual preference that differs from the ideal preference they desire to hold^4–6^ (For more information about actual-ideal preference discrepancies, including why they emerge and their distinction from related constructs, see Supplementary Information). People often struggle to do what they ideally want as a result of this conflict between their actual and ideal preferences^6,7^. For instance, someone might *ideally* want to read even-handed, well-researched political reporting, but she might *actually* be drawn to something more sensationalized like political opinion clickbait.

Although temptations like these have always hindered people from acting in line with their ideal preferences, recommendation algorithms may in fact be making people's struggles even worse. This is because the recommendation algorithms currently used by technology platforms are designed to maximize user engagement, such as clicks, likes, and shares^1^. Targeting actual preferences could increase user engagement (e.g., clicks) because people find it harder to resist such content^8^. However, this approach might not make people feel better off or that their time has been well spent. It may also reduce their goodwill toward the company providing the recommendation. In contrast, targeting people's ideal preferences could generate somewhat fewer clicks but could benefit both users and companies in other more meaningful ways.

To examine this empirically, we built algorithmic recommendation systems that generated real-time, personalized recommendations specifically tailored to either a person's actual or ideal preferences. Then, in a high-powered, pre-registered experiment, we measured the effects that these algorithmic recommendation systems had on individuals' perceptions of the recommendation, perceptions of the company, and reactions after reading the recommended content.

## Results

**Figure 1:**
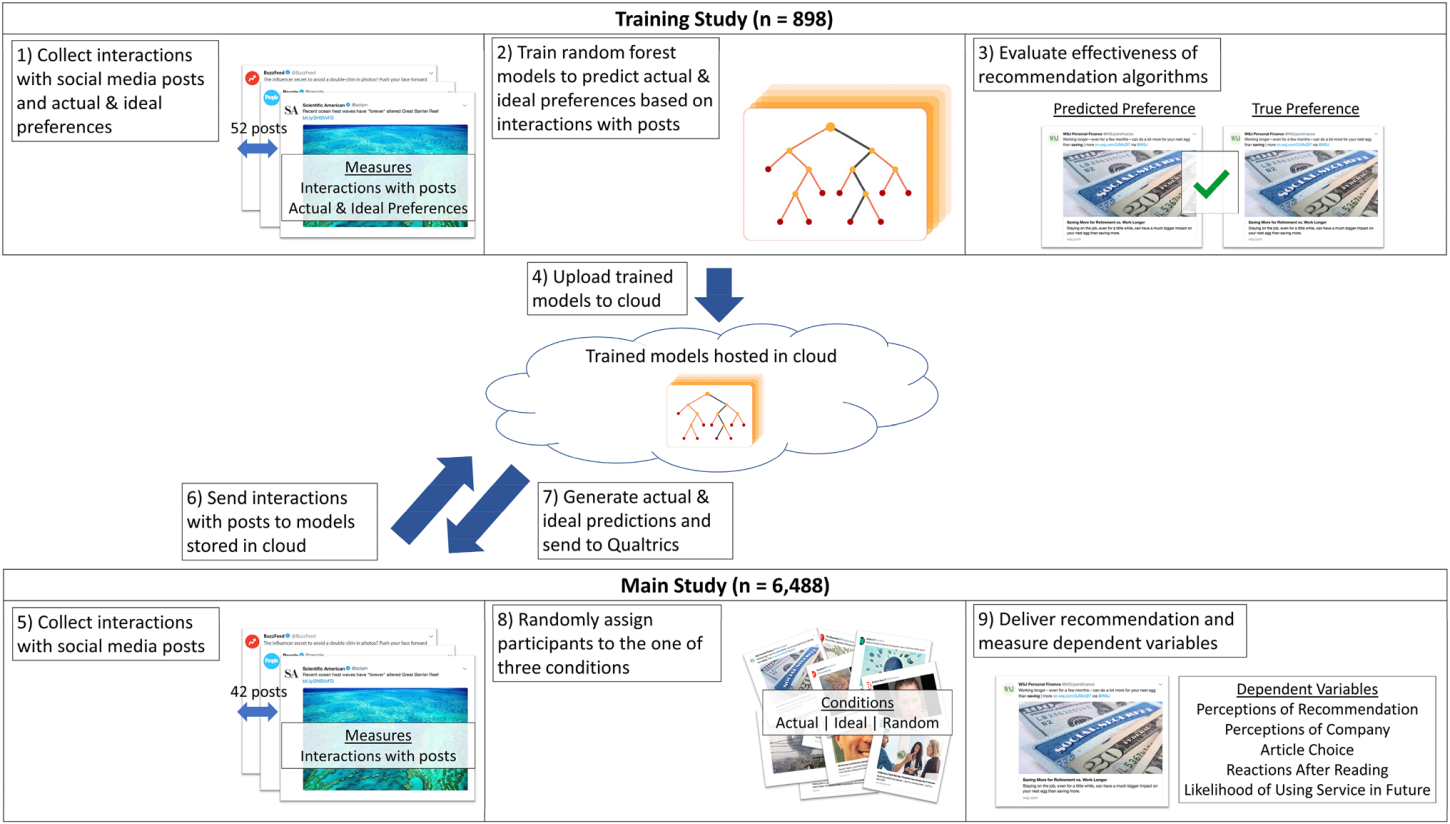
Research pipeline. In the training study, we collected data to train models that predicted people's actual and ideal preferences based on their interactions with social media posts of news articles. Trained models were hosted in the cloud. In the main study, we used these models to predict the actual and ideal preferences of new participants. Based on our predictions, we delivered personalized recommendations for news articles to these participants and measured their responses.

An overview of our research pipeline is shown in Fig. 1. We first trained machine learning models to predict people's actual and ideal preferences for digital content from their interactions with other digital content. We used these models to construct algorithmic recommendation systems. We then tested the effects of these recommendation systems in a pilot study and ran a power analysis to determine the measures and sample size^9^ for our main study, which we pre-registered (Pre-registration is available on the Open Science Framework: https://osf.io/nzbxy/?view_only=1c05ace934ea4ef4bd64342283fed926).

The purpose of the training study (*n* = 898) was to collect the data necessary to build our algorithmic recommendation systems. In the training study, participants interacted with 52 posts of news articles taken from a prominent social media platform. In addition, they indicated their *actual* (“How much do you ACTUALLY want to read this article?”) and *ideal* (“How much would you IDEALLY want to read this article?”) preferences for each article on a 100-point scale.

Using these data, we trained random forest models to predict how much a particular individual would actually and ideally want to read each of 10 of these articles based on their interactions (i.e., participants indicated their inclination to read each article now as well as their inclination to save each article for later using a reading list feature) with the other 42 articles.

To evaluate how well the models would perform on a sample of new participants, we used 10-fold cross-validation on the 898 participants in the training study. In this approach, participants were randomly divided into 10 subsets. For each subset, we trained models on the 90% of the data not in the subset and tested these models on the remaining 10% of participants in the subset. Importantly, all of the predictions for a given individual were generated from models trained on other people. This prevented over-fitting (i.e., models that overestimate accuracy by training and testing on the very same data). Using this approach, we could estimate how well the models we trained would perform on a new sample of participants, such as those in the main study.

We performed several tests to evaluate the effectiveness of our algorithms. In particular, we verified the accuracy of our models, confirmed that our personalized predictions performed better than a non-personalized approach, and established that our intended experimental manipulations would effectively and uniquely target the constructs they were designed to (see “Methods” section for details).

After validating the machine learning models, we developed an algorithmic recommendation system that used these models to generate real-time recommendations tailored to either a person’s actual or ideal preferences. Specifically, participants would be able to interact with social media posts, and these interactions would be relayed to our machine learning models stored in Google Cloud, which would return predictions about which new content each particular participant would most actually or ideally want to read (see “Methods” section for more details).

We then conducted a pre-registered study to examine how people perceive and respond to such recommendations (*n* = 6488). Sample size was determined based on a pilot study (*n* = 958).

In the main pre-registered study, new participants first interacted with 42 posts of news articles from the training study. Based on only these interactions, our recommendation algorithms used the machine learning models from the training study to predict these participants' actual and ideal preferences for the 10 articles they had not seen. These predictions enabled us to generate a personalized article recommendation for each participant. The study employed three conditions. Each participant in the actual (or ideal) condition was recommended a news article from social media based on what our models predicted this particular individual would actually (or ideally) most want to read. Participants in the control condition were recommended a randomly selected article.

For our dependent variables, we asked participants for their perceptions of the recommendation and the recommendation service (see Table 1 for full text and pre-registered predictions for all dependent variables). For the final portion of the study, participants had the option to click to read the recommended article. Regardless of their choice, all participants subsequently read the recommended article and reported their reactions to avoid self-selection effects. Although this approach addresses the issue of self-selection, it may be less ecologically valid, as people on digital platforms typically only read the articles they have chosen to read. To address this potential challenge to ecological validity, as pre-registered, we examined whether any of our subsequent results differed between those who had organically chosen to read the article they received and those who had not. The choice to read the recommended article did not interact with any of our significant results.

**Table 1:** Dependent variables.

Question Category	Question	Pre-registered Prediction	Prediction Supported by Data
Perceptions of Recommendation	How helpful is this recommendation?	Ideal will be higher than actual	Yes
	To what extent would receiving this recommendation make you better off?	Ideal will be higher than actual	Yes
Perceptions of the Company Generating the Recommendation	To what extent does the company making this recommendation seem to have your best interest at heart?	Ideal will be higher than actual	Yes
	How much would you be willing to pay for this recommendation service per month? (Free response)	Ideal will be higher than actual	Yes
Article Choice	For the next stage of this study, you will be required to read an article. You can either choose the article recommended to you, or we will randomly choose an article for you from a set of ten articles. What would you like to do? (Binary forced choice)	Actual will be higher than ideal	Yes
Reactions After Reading the Recommended Article	How much did you like reading this article?	Actual will be higher than ideal	Yes
	How enjoyable was reading this article?	Actual will be higher than ideal	Yes
	To what extent do you feel that reading this article made you better off?	Ideal will be higher than actual	Yes
	How likely would you be to read similar articles in the future?	Actual will be higher than ideal	No
	To what extent do you feel your time was well spent reading this article?	Ideal will be higher than actual	Yes
Likelihood of Using Recommendation Service in the Future	After having read this article, how likely would you be to use this recommendation service in the future?	Ideal will be higher than actual	Yes

All questions were answered on a 7-point scale, except for willingness to pay and article choice. The order of questions was randomized within category.

An initial analysis revealed that the personalized recommendations in both the actual and ideal conditions performed significantly better on all dependent variables than the random recommendations in the control condition (see Table S2 in Supplementary Information).

**Figure 2:**
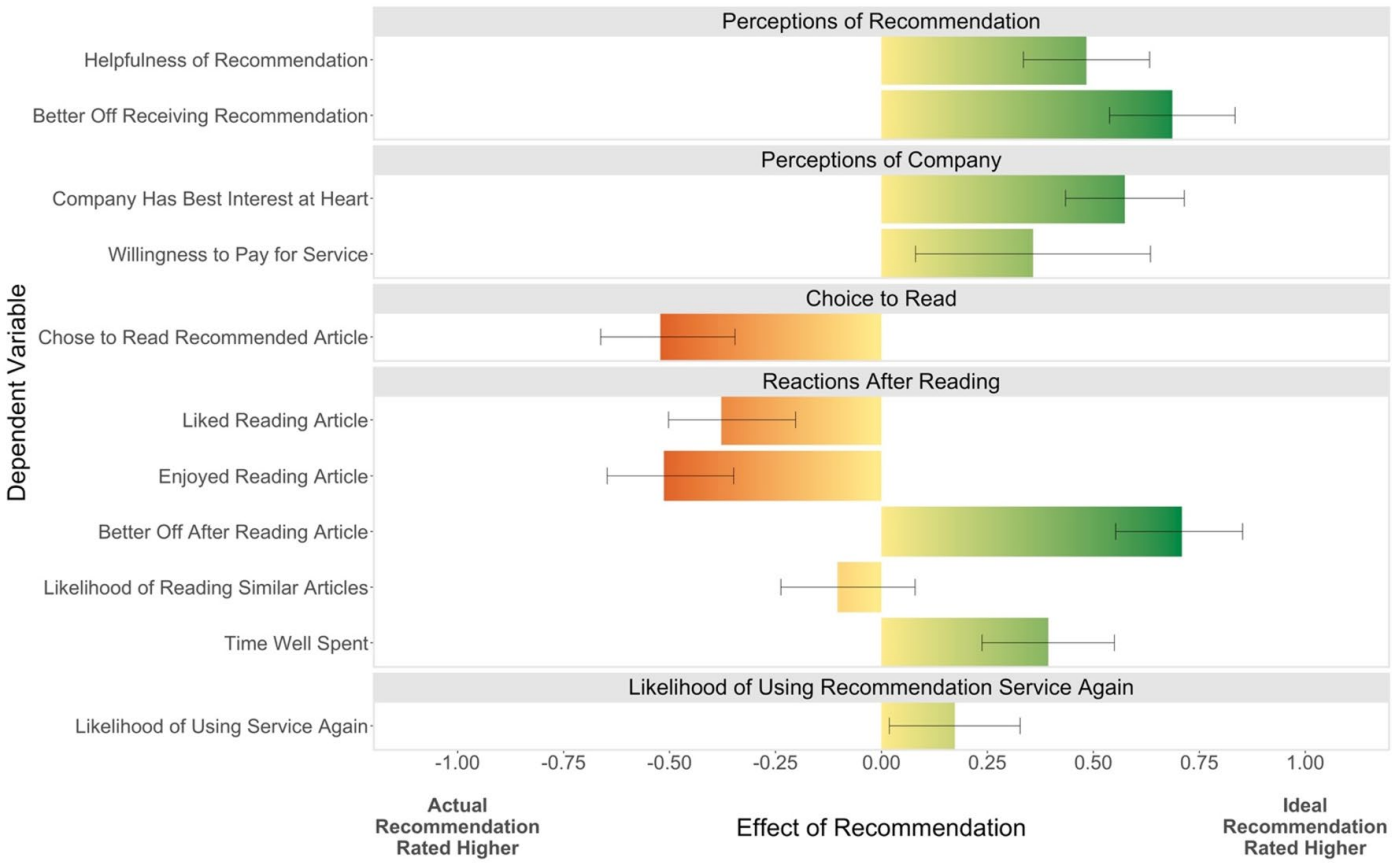
Effect of receiving recommendation tailored to ideal (vs. actual) preferences. Bars correspond to the effect of receiving a recommendation tailored to ideal (vs. actual) preferences when these recommendations differed (*n* = 2504). For continuous measures, unstandardized linear regression coefficients are presented; for the sole dichotomous measure, choosing to read the recommended article, the unstandardized logistic regression coefficient is presented. Error bars correspond to 95% confidence intervals.

In our primary analysis, we compared the effect of receiving a recommendation tailored to ideal versus actual preferences when these recommendations differed (*n* = 2504). We expected that tailoring recommendations to actual preferences (vs. ideal preferences) would increase engagement but negatively affect other important outcomes, such as whether people felt better off or believed that the company had their best interest at heart (see Table 1). Overall, 10 out of our 11 pre-registered predictions were supported by the data. Results are presented in Fig. 2.

Catering algorithmic recommendations to actual rather than ideal preferences did increase engagement, as people were more likely to click to read the recommended article (52% vs. 40%, *p* < 0.001). Participants given actual-preference recommendations also reported enjoying (*M*_*actual*_ = 4.02, *M*_*ideal*_ = 3.52, *t* = − 6.54, *p* < 0.001) and liking (*M*_*actual*_ = 4.12, *M*_*ideal*_ = 3.77, *t* = − 4.61, *p* < 0.001) reading the article more.

Nonetheless, tailoring recommendations to ideal preferences provided users with several meaningful benefits. Even before choosing whether to read the recommended article, those who saw an ideal-preference (vs. actual-preference) recommendation rated the recommendation as more helpful (*M*_*actual*_ = 3.83, *M*_*ideal*_ = 4.32, *t* = 6.37, *p* < 0.001) and felt that receiving this recommendation would make them better off (*M*_*actual*_ = 3.37, *M*_*ideal*_ = 4.06, *t* = 9.10, *p* < 0.001). In addition, they also felt better off after reading the recommended article (*M*_*actual*_ = 3.20, *M*_*ideal*_ = 3.91, *t* = 9.19, *p* < 0.001) and felt that their time was well spent (*M*_*actual*_ = 3.87, *M*_*ideal*_ = 4.26, *t* = 4.94, *p* < 0.001).

Importantly, beyond its benefits for users, targeting ideal rather than actual preferences also showed potential benefits for companies. Ideal recommendations increased how much individuals were willing to pay for the service (*M*_*actual*_ = 1.84, *M*_*ideal*_ = 2.19, *t* = 2.53, *p* = 0.012), how much they felt the company had their best interest at heart (*M*_*actual*_ = 3.43, *M*_*ideal*_ = 4.00, *t* = 8.04, *p* < 0.001), and their inclination to use the service again (*M*_*actual*_ = 3.30, *M*_*ideal*_ = 3.48, *t* = 2.20, *p* = 0.028).

## Discussion

These results suggest that actual-preference and ideal-preference recommendations have different strengths. Imagine a person who actually wanted to read a sensationalized political opinion piece but ideally wanted to read even-handed political reporting. Our results suggest that if she were shown an algorithmic recommendation for what she actually (vs. ideally) wanted, she would be more likely to give in to her temptation to read the sensationalized political opinion piece and would even enjoy reading it more. However, after consuming this content, she would not feel that her time was as well spent compared to if she had read the even-handed political reporting. Relatedly, she would not feel that the recommendation for the sensationalized political opinion was as helpful as a recommendation for even-handed political reporting would have been, and she would be less likely to feel that the company generating the recommendation has her best interests at heart. Perhaps most interestingly, even though she would be more likely to choose to read the sensationalized opinion and enjoy reading it more, she would not feel better off and would be less willing to pay for the service and less inclined to use the service again in the future.

The actual and ideal recommendation algorithms we developed recommended different kinds of content, reflecting differences in people’s actual versus ideal preferences. For instance, in our primary sample, the algorithm trying to optimize for people’s ideal preferences was more likely to recommend an article with personal finance tips than an article revealing a salacious paternity scandal. The opposite was true for the algorithm optimizing for people’s actual preferences. By recommending different types of content, these between-condition differences in articles recommended led to between-condition differences in the outcomes we observed.

Given this evidence, tailoring algorithms for ideal rather than actual preferences is not a strictly dominant approach, but it does offer certain advantages. Both digital platforms and their users may ultimately stand to benefit if there were more options to incorporate ideal preferences into algorithmic recommendations.

Social media companies generally place great emphasis on increasing user engagement. Scholars have previously theorized that this may not be best for users^1,10^. Our research provides empirical evidence that although targeting actual rather than ideal preferences leads to higher engagement, this does not maximize the extent to which users feel better off. Indeed, people are more likely to engage with (i.e., share) content that triggers strong emotions, such as anger, anxiety, or disgust^11–13^, yet the widespread distribution and consumption of such content is not necessarily better for the sharers, recipients, or society at large.

Given the issues that result from overly prioritizing engagement, various solutions have been proposed to this problem. Some have suggested restricting people’s exposure to social media, such as by imposing time limits^14–16^. A different approach suggests that companies move beyond observable engagement metrics to incorporate latent constructs, such as a person’s desires^17^. A theoretical model has been developed to account for the fact that, because people have preference inconsistencies, they might end up consuming content on social media even though they derive very little utility from it^18^. This theoretical model suggests that it should be possible to modify recommendation algorithms to provide people with content that increases their utility. We complement these ideas by presenting empirical evidence that it is possible to train algorithms to optimize for what each person ideally wants and show that this can have valuable benefits.

Our research also contributes to the literature on the psychology of algorithms^19–21^ by examining how people perceive and respond to algorithmic recommendations. Even when people actually and ideally want different things, we show that people are more inclined to reward recommendation algorithms that cater to their ideal preferences.

More broadly, the current research has implications for the burgeoning topic of AI alignment. In the coming years, as computers become increasingly tasked with making consequential decisions, it is imperative that the objectives machines pursue are aligned with human preferences and values^10^. This is further complicated by the fact that humans themselves frequently have conflicting preferences (e.g., actual vs. ideal). Effective AI systems must be able to learn the multifaceted and, at times, inconsistent nature of human preferences to avoid producing outcomes that are aligned with one aspect of people’s preferences but neglect another. This research contributes to this endeavor by training AI systems to model theoretically distinct human preferences that have been widely discussed in the behavioral sciences^22^, but have received somewhat less attention in the artificial intelligence community.

In addition to behavioral science informing artificial intelligence, our research also shows how artificial intelligence can inform behavioral science. A growing movement in public policy has focused on nudging people toward actions that are better for them (e.g., saving more for retirement)^23,24^. However, nudges are usually not personalized and require a choice architect to decide what is best^25^. This could be problematic as different people may have different ideals. Our research shows that artificial intelligence can learn what different people ideally want and deliver personalized recommendations catered to each person's unique ideal preferences. Incorporating such technology into nudges could therefore help people live better lives based on their own definitions of what a “better” life means to them.

Though our findings suggest that companies could foster goodwill and other benefits by placing more weight on ideal preferences, they may be resistant to making this change. Their current business models profit from engagement, and as we show, targeting ideal rather than actual preferences results in somewhat lower engagement. Therefore, it may be necessary to provide the right incentives to better align the interests of social media platforms with societal welfare. As a first step, policymakers could incentivize companies to provide more transparency over what their recommendation algorithms are programmed to optimize for and give users more control over these parameters. For instance, they could allow individuals to set the frequency at which the algorithm suggests specific kinds of content (e.g., individuals could explicitly request more recommendations for retirement savings and less for sensationalized celebrity gossip, even if their clicks might suggest otherwise). Ultimately, both individuals and companies could benefit if the recommendations people receive are more closely aligned with who they ideally want to be.

## Methods

### Main study

Our methods and analysis plan were pre-registered on OSF (Open Science Framework) prior to data collection. The pre-registration is available at the following url: https://osf.io/nzbxy/?view_only=1c05ace934ea4ef4bd64342283fed926. All methods were carried out in accordance with relevant guidelines and regulations, and all experimental protocols were approved by the Stanford University Institutional Review Board (IRB). Study participants provided informed consent before taking part in the study.​

Below, we provide a description of the study design and our analysis strategy.

#### Study design

##### Consent and attention check warning

First, participants were asked to consent to participate in the survey. They were also informed that they would only receive payment if they correctly answered all attention check questions.

##### Demographics and news article preferences

Next, we collected the following demographic information from participants: gender, age, household income, political preferences, and religious views. We also asked participants to indicate how much they like reading news articles, how often they read news articles, and how much they like reading each of the following types of news articles: Technology, Business, Health, US Politics, International Politics, Celebrity Gossip, Lifestyle, New York Times, Buzzfeed, Wall Street Journal, The Blaze, Breitbart News, Entertainment Weekly, People Magazine, US Weekly, Perez Hilton, Fox News, Huffington Post, CNBC, CNN, The Economist, The New Yorker, Slate, US News and World Report, Reader's Digest, Harvard Business Review, BBC World News, NPR, How Stuff Works, TechCrunch, Wired, and Al Jazeera News. As pre-registered, these initial questions were included for purely exploratory purposes. They were not used as inputs in the machine learning model. However, we did test whether our main findings held when controlling for these additional measures.

##### Collecting participant preferences to generate real-time machine learning predictions

Participants then saw 42 posts of news articles and were asked to indicate how likely they would be to read the article now and how likely they would be to save the article to a reading list to read later. Both questions were asked on a 100-point slider scale with poles labeled “Not at all likely” and “Very likely.” The default choice was the midpoint, and all questions required a response. Participants were asked to assume each article would take approximately two minutes to read. All 42 posts can be found in the document called “Article Posts Used to Train Model” in our OSF directory, available here: https://osf.io/rvjns/?view_only=a3b9778262dc4ee9a3edf3285d5647a5. An example item with the rating scales participants used can be found in the document titled “Example Item Used to Train Model” in our OSF directory. Each post was presented on its own page.

We then presented participants with three attention check questions. These questions asked participants whether they had been shown three particular posts. In our analyses, as pre-registered, we only included participants who correctly answered all three attention check questions.

##### Generating personalized recommendations using real-time, cloud-based machine learning

After participants finished interacting with the 42 posts, their responses were automatically sent from Qualtrics to Google Cloud. On Google Cloud, each participant’s ratings were input into algorithms we created that used machine learning to predict each individual’s actual and ideal preferences. These algorithms were trained on data previously collected from 898 other individuals in an initial training study (See “Training Study” in “Methods” section for more details).

Based on each participant’s ratings, the algorithms determined which of 10 previously unseen articles each participant was most inclined to *actually* want to read and which of these 10 articles each participant was most inclined to *ideally* want to read. The posts for these 10 previously unseen articles can be found in the document titled “Article Posts Used for Recommendations” in our OSF directory. The models' predictions regarding each individual’s preferences were relayed back to Qualtrics to determine which recommendation this participant would receive.

##### Random assignment to condition

In Qualtrics, we randomly assigned participants to one of three conditions: actual, ideal, or random. This determined which article participants would be recommended from a set of 10 possible articles. In the actual condition, participants were recommended the article that we predicted they actually wanted to read most from this set. In the ideal condition, participants were recommended the article that we predicted they ideally wanted to read most from this set. In the random condition, participants were recommended a randomly selected article from this set.

##### Recommendation and dependent variables

We then showed participants a post of the article recommended to them and asked them to answer several questions. Prior to data collection, we pre-registered directional predictions for all of these questions based on the results of a pilot study. Table 1 contains the full text of all questions and their corresponding categories. It also contains our pre-registered predictions for all questions and their corresponding outcomes.

Questions were answered on a 7-point scale, except for willingness to pay and article choice. As pre-registered, prior to analysis, we Winsorized willingness to pay to a maximum of $15 per month. This is because many customers appear unwilling to pay anything for a recommendation service alone (almost half of the participants in a pilot study reported $0 for this measure). Moreover, for those who are willing to pay for such a service, it appears unlikely that they would be willing to pay significantly more for this service than for comparable services at the time of the study that not only recommend articles but also provide access to article content, such as *Apple News Plus* ($9.99 per month) or the *New York Times Digital Subscription* ($9.99–$15.99 per month). The order of questions was randomized within category.

Upon receiving the recommendation, participants first answered several questions about their perceptions of the recommendation and their perceptions of the company generating the recommendation (i.e., Helpfulness of Recommendation, Better Off Receiving Recommendation, Company Has Best Interest at Heart, Willingness to Pay for Service).

Participants were then asked whether they wanted to read the article they had been recommended. Regardless of their choice, all participants read the recommended article to prevent self-selection effects. We also conducted analyses to evaluate whether this choice affected any of the subsequent results (see “Data collection and analysis” section below). After participants finished reading the article, we asked them additional questions about their reactions.

#### Data collection and analysis

We targeted a sample size of 6500 new participants using TurkPrime. This sample size was determined by conducting power analyses on data from a pilot study (*n* = 958). As pre-registered, we excluded participants who failed any of the three attention checks, did not finish the survey, or took the survey more than once. The final sample size was 6488 participants (54.8% female, *M*_*age*_ = 36.19, *SD*_*age*_ = 11.9).

For our initial analysis, we compared both the actual and ideal conditions to the random condition using linear regression models, as pre-registered. This allowed us to compare the effects of both kinds of customized recommendations to the effects of random (uncustomized) recommendations. Summary statistics are presented in Table S1, and regression results are presented in Table S2. Recommendations tailored to either actual or ideal preferences outperformed random recommendations on all measures. This provides evidence that our algorithms were successfully inferring participants’ preferences based on their ratings of other articles (for further evidence of the effectiveness of our algorithms, see “Training Study” in “Methods” section).

After demonstrating that customized recommendations were superior to random recommendations, in our primary analysis, we compared the actual and ideal conditions to each other. Specifically, we examined which type of recommendation would perform better for each dependent variable. Consistent with prior work^5^, participants did not always have a discrepancy between their actual and ideal preferences. As a result, sometimes the article a participant would have been recommended in the actual condition was the same as the article the participant would have been recommended in the ideal condition. In such cases, because the article recommended was the same regardless of condition, there was no experimental manipulation between the actual and ideal conditions. As it is not meaningful to test for between-condition differences when both conditions are the same, we focused on the instances in which they were discrepant. Specifically, to compare the actual and ideal conditions, as pre-registered, we examined only participants in these conditions who would have received a different recommendation had they been in the other condition (*n* = 2504).

Based on the results of our pilot study, we had pre-registered directional predictions and statistical analyses for all dependent variables. Overall, 10 out of 11 of our pre-registered predictions were supported by the data. Summary statistics and significance tests are provided in Table S3. All of the t-tests reported in our studies were two-tailed.

Although not pre-registered, we conducted additional analyses to determine whether these results held when controlling for the collected demographic variables, news preferences, and news consumption frequency. Results are shown in Table S4. All patterns remained consistent, even when including these controls.

Furthermore, to avoid self-selection effects, we ensured that everyone in our study read the recommended article (even those who indicated they would not choose to read the article on their own). Though this approach addresses the issue of self-selection, we acknowledge that it may be less ecologically valid as users on digital platforms typically only read the articles they have chosen to read. Thus, to address these concerns regarding ecological validity, we examined whether the choice to read the recommended article affected any of our subsequent results. In particular, we analyzed our data separately for those who had chosen to read the recommended article (as would typically be the case on social media; See Table S5) and those who had not (See Table S6). We also tested for any interactions between choosing to read the recommended article and condition (actual vs. ideal) on the post-reading measures (See Table S7). All of these decisions regarding the experimental design and analysis were made prior to data collection and are documented in our pre-registration.

We found that the choice to read the recommended article did not interact with any of our significant results. The only interaction that emerged between condition (actual vs. ideal) and choosing to read the recommended article was on one’s reported likelihood of reading similar articles in the future (*b* = − 0.29, 95% CI [− 0.58, 0.00], *t*(2499) = − 1.96, *p* = 0.050), which was at the threshold of conventional significance. For participants who chose to read the article, there was no significant effect of condition on reported likelihood of reading similar articles in the future (*b* = − 0.02, 95% CI = [− 0.23, 0.20], *t*(2499) = − 0.14, *p* = 0.889). However, for participants who did *not* choose to read the article, there was a significant effect of condition, such that participants who received a recommendation tailored to their ideal (vs. actual) preferences reported a higher likelihood of reading similar articles in the future (*b* = 0.27, 95% CI = [0.08, 0.47], *t*(2499) = 2.75, *p* = 0.006). Although we had no a priori predictions about interactive effects, this result suggests that individuals who would not organically choose to follow an ideal-preference recommendation but do in fact follow this recommendation may be more likely to seek out similar content in the future.

### Training study

#### Study design

In order to train machine learning models to predict people’s preferences, we required training data, which we acquired by conducting the training study. The purpose of this training study was to allow us to build models that predicted people’s preferences for a particular article based on their interactions with other content and to verify that these models were sufficiently accurate for use in an experimental manipulation. The initial study used a similar design to the main study with the following differences.

First, participants were not given a recommendation to evaluate as this study was designed to help us determine which recommendations we would give participants in the main study.

Second, participants rated 52 posts of news articles rather than 42, 10 of which were selected as the possible recommendations in the main study based on size of the preference discrepancies they evoked.

Third, in addition to indicating how likely they would be to read each article now and how likely they would be to save each article to read later, participants also indicated how much they actually and ideally wanted to read each article.

#### Data collection and analysis

We recruited 1000 participants for the initial study and excluded those who failed any of the attention checks, leaving a sample of 898 individuals. Using these data, we trained random forest models to predict how much an individual actually or ideally wanted to read an article based on their interactions with other articles. In particular, we predicted participants’ actual and ideal preferences for the 10 possible articles in the recommendation set using their “read now” and “read later” scores for the remaining 42 articles.

To train the models, we employed the random forest algorithm. This algorithm uses multiple decision trees to generate predictions and is able to teach itself complex relationships, such as those involving non-linearity and high-order interactions, without the need to explicitly model these phenomena^26^. For each of the 10 articles in the recommendation set, we trained two separate random forest models: one to predict how much a participant would actually want to read the article and another to predict how much this participant would ideally want to read the article.

As these recommendations would be used for the experimental manipulation in the main study, we wanted to verify that the models were accurately predicting people’s preferences, performing better than a non-personalized approach, and effectively discriminating between the actual and ideal dimensions. To do so, we conducted the following analyses.

To test the accuracy of our models, we used 10-fold cross-validation. We calculated the root mean square error and mean absolute error of our predictions as compared to the true values for both actual (*RMSE* = 29.93, *MAE* = 25.15) and ideal preferences (*RMSE* = 29.52, *MAE* = 24.36). In effective terms, our results showed that at least 55% of the time, the article predicted by the models to be highest on actual or ideal ratings was in the participant’s top three articles on that dimension (see Figs. S1 and S2 for the full distributions). Note that this accuracy is likely an underestimate of the true accuracy that could be achieved by artificial intelligence. With the size and richness of data and computational resources available to most technology companies, it seems probable that models could achieve notably higher accuracies. Such recommendation algorithms would likely have even larger effect sizes than those we observed in our main study.

Next, we evaluated whether our personalized predictions performed better than a non-personalized approach, in which everyone would have been recommended the same article (i.e., giving everyone the article that was rated highest on average along the actual or ideal dimensions). A paired t-test revealed that participants reported higher actual preferences for the article our personalized models predicted would be the highest on the actual dimension (*M* = 59.10, *SD* = 33.41) than for the non-personalized overall highest rated article on the actual dimension (*M* = 52.47, *SD* = 34.42), mean difference (*M*_*d*_) = 6.62, 95% *CI* [4.57, 8.68], *t*(897) = 6.33, *p* < 0.001. In addition, the same was true for the ideal dimension— a paired t-test showed that participants’ ideal preferences were higher for the article our personalized models predicted would score highest on the ideal dimension (*M* = 68.31, *SD* = 31.55) than for the non-personalized overall highest rated article on the ideal dimension (*M* = 65.14, *SD* = 33.47), mean difference (*M*_*d*_) = 3.17, 95% *CI* [1.65, 4.68], *t*(897) = 4.10, *p* < 0.001.

Additionally, we wanted to ensure that our experimental manipulation was effectively and uniquely targeting the construct it was designed to. To examine this, we first subsetted on participants who would have been included in our experimental analyses, i.e., those who would have received a different recommendation in the actual vs. ideal condition. We then simulated the recommendations these participants would have received in the main study and evaluated how effective these recommendations would be in uniquely targeting actual and ideal preferences.

First, between conditions, we compared the articles participants would have received to each other. A paired t-test revealed that participants’ actual preferences were higher for the article they would have received in the actual condition (*M* = 56.53, *SD* = 35.08) than the article they would have received in the ideal condition on the actual dimension (*M* = 52.09, *SD* = 33.18), mean difference (*M*_*d*_) = 4.44, 95% *CI* [0.97, 7.91], *t*(447) = 2.52, *p* = 0.012. Similarly, the article participants would have received in the ideal condition had higher associated ideal preferences (*M* = 65.06, *SD* = 33.22) than the article they would have received in the actual condition (*M* = 50.31, *SD* = 36.31), *M*_*d*_ = 14.76, 95% *CI* [11.11, 18.40], *t*(447) = 7.95, *p* < 0.001.

Second, within each condition, we compared actual and ideal preferences for the article participants would have been recommended. We found that for the article that would have been recommended in the actual condition, the reported actual preferences (*M* = 56.53, *SD* = 35.08) were higher than the reported ideal preferences (*M* = 50.31, *SD* = 36.31), *M*_*d*_ = 6.22, 95% *CI* [3.34, 9.09], *t*(447) = 4.25, *p* < 0.001. Similarly, for the article that would have been recommended in the ideal condition, the reported ideal preferences (*M* = 65.06, *SD* = 33.22) were higher than the reported actual preferences (*M* = 52.09, *SD* = 33.18), *M*_*d*_ = 12.98, 95% *CI* [10.29, 15.66], *t*(447) = 9.50, *p* < 0.001.

Taken together, these results suggest that the models used by our recommendation algorithms were effectively targeting participants’ actual and ideal preferences. Based on these results, we used these algorithms in the main study to generate real-time, personalized recommendations targeted to either participants’ actual or ideal preferences.

## Supplementary Information

Supplementary Information.

## Data Availability

Data and materials are available at: https://osf.io/rvjns/?view_only=a3b9778262dc4ee9a3edf3285d5647a5.

## References

[1] Alter AL (2017). Irresistible: The Rise of Addictive Technology and the Business of Keeping us Hooked.

[2] United States Congress. Protecting Kids Online: Testimony from a Facebook Whistleblower. (2021).

[3] Allcott H, Braghieri L, Eichmeyer S, Gentzkow M (2020). The welfare effects of social media. Am. Econ. Rev..

[4] DeMarree KG, Clark CJ, Wheeler SC, Briñol P, Petty RE (2017). On the pursuit of desired attitudes: Wanting a different attitude affects information processing and behavior. J. Exp. Soc. Psychol..

[5] DeMarree KG, Christian Wheeler S, Briñol P, Petty RE (2014). Wanting other attitudes: Actual-desired attitude discrepancies predict feelings of ambivalence and ambivalence consequences. J. Exp. Soc. Psychol..

[6] Maio GR, Thomas G (2007). The epistemic-teleologic model of deliberate self-persuasion. Personal. Soc. Psychol. Rev..

[7] Wheeler, S. C. & DeMarree, K. G. Prevalence, antecedents and consequences of actual-desired attitude discrepancies. in *Handbook of Research on Identity Theory in Marketing* 346–359 (Edward Elgar Publishing, 2019). 10.4337/9781788117739.00035.

[8] Milkman KL, Rogers T, Bazerman MH (2009). Highbrow films gather dust: Time-inconsistent preferences and online DVD rentals. Manage. Sci..

[9] Sakaluk JK (2016). Exploring Small, Confirming Big: An alternative system to The New Statistics for advancing cumulative and replicable psychological research. J. Exp. Soc. Psychol..

[10] Russell, S. *Human Compatible: Artificial Intelligence and the Problem of Control*. (2019).

[11] Berger J, Milkman KL (2012). What makes online content viral?. J. Mark. Res..

[12] Milkman KL, Berger J (2014). The science of sharing and the sharing of science. Proc. Natl. Acad. Sci..

[13] Heath C, Bell C, Sternberg E (2001). Emotional selection in memes: The case of urban legends. J. Pers. Soc. Psychol..

[14] Saig, E. & Rosenfeld, N. Learning to Take a Break: Sustainable Optimization of Long-Term User Engagement. (2022).

[15] Lyngs U (2019). Self-control in cyberspace: Applying dual systems theory to a review of digital self-control tools. Conf. Hum. Factors Comput. Syst. Proc..

[16] Allcott H, Gentzkow M, Song L (2022). Digital addiction. Am. Econ. Rev..

[17] Ekstrand, M. D. & Willemsen, M. C. Behaviorism is not enough: Better recommendations through listening to users. *RecSys 2016 Proc. 10th ACM Conf. Recomm. Syst.* (2016) 10.1145/2959100.2959179.

[18] Kleinberg, J., Mullainathan, S. & Raghavan, M. The Challenge of Understanding What Users Want: Inconsistent Preferences and Engagement Optimization. *arXiv Prepr.* (2022).

[19] Dietvorst BJ, Simmons JP, Massey C (2018). Overcoming algorithm aversion: People will use imperfect algorithms if they can (even slightly) modify them. Manage. Sci..

[20] Logg, J. M. The psychology of Big Data: Developing a “theory of machine” to examine perceptions of algorithms. in *The psychology of technology: Social science research in the age of Big Data.* 349–378 (American Psychological Association, 2022). 10.1037/0000290-011.

[21] Fast NJ, Jago AS (2020). Privacy matters… or does It? Algorithms, rationalization, and the erosion of concern for privacy. Curr. Opin. Psychol..

[22] Milkman KL, Rogers T, Bazerman MH (2008). Harnessing our inner angels and demons: What we have learned about want/should conflicts and how that knowledge can help us reduce short-sighted decision making. Perspect. Psychol. Sci..

[23] Thaler RH, Sunstein CR (2008). Nudge: Improving Decisions About Health, Wealth, and Happiness.

[24] Benartzi S (2017). Should governments invest more in nudging?. Psychol. Sci..

[25] Mills S (2020). Personalized nudging. Behav. Public Policy.

[26] Grömping U (2009). Variable importance assessment in regression: Linear regression versus random forest. Am. Stat..

