# Supplementary material for: Tailoring recommendation algorithms to ideal preferences makes users better off
Source: Sci Rep. 2023 Jun 8;13:9325. doi: 10.1038/s41598-023-34192-x (PMC10250302; doi:10.1038/s41598-023-34192-x)
Supplement: Supplementary file 1 — Supplementary Information. [file 41598_2023_34192_MOESM1_ESM.pdf]

Supplementary Information

Why Actual-ideal Preference Discrepancies Emerge

Distinction Between Actual-ideal Preference Discrepancies and Related Constructs

Timing Analyses

Fig. S1 – S2

Fig. S1. True rank of article predicted to be highest (actual).

Fig. S2. True rank of article predicted to be highest (ideal).

Table S1 – S7

Table S1. Summary statistics for all conditions and all participants.

Table S2: Regression results for all conditions and all participants.

Table S3. Summary statistics and significance tests comparing the actual and ideal conditions for participants with a preference discrepancy.

Table S4. Regression results comparing the actual and ideal conditions for participants with a preference discrepancy with controls.

Table S5. Summary statistics and significance tests for post-reading reactions of participants who chose to read the recommended article.

Table S6. Summary statistics and significance tests for post-reading reactions of participants who chose not to read the recommended article.

Table S7. Interaction of condition and choice to read recommended article on post-reading dependent variables.

References

Why Actual-ideal Preference Discrepancies Emerge

The reader may be curious about why a person may have an ideal attitude that is different from their actual attitude. This is to ask two questions: (1) why would a person desire to have any particular attitude, and (2) why would that person not just adopt the attitude they desire (i.e., make their actual attitude match their ideal attitude). Many potential reasons one might desire to hold a particular attitude involve achieving an ideal state or outcome. For example, consistency theories (e.g., Festinger, 1957; Heider, 1958) suggest that many people desire to hold attitudes that are consistent with their actions and with other held attitudes. Likewise, people desire to hold attitudes that are congruent with those of important liked others, as not doing so can create intrapsychic conflict in the form of subjective ambivalence (Priester & Petty, 2001). People may also desire attitudes because they are useful in facilitating goal achievement (e.g., DeMarree, Clark, Wheeler, Briñol, & Petty, 2017; Ferguson, 2007, 2008; Fitzsimons & Shah, 2008). That is, goal progress is more likely when one likes activities, objects, and other people that facilitate goal progress and dislikes those that serve as obstacles to goal progress. Holding particular attitudes (e.g., liking watching sports or other popular entertainment) can facilitate social connection because it promotes shared consumption and subsequent conversation. Holding certain attitudes can also help facilitate identity expression (Wheeler & Bechler, 2020). Liking things consistent with an ideal individual identity (e.g., academic or sophisticate) or group affiliation (e.g., American) help align one toward such identities and convey them to others. Last, and most simply, people prefer liking things to disliking them (e.g., the attraction or positivity effect; Jordan, 1953). A person is likely to prefer liking their apartment, job, and spouse to disliking them, for example.

The second question concerns why one wouldn’t simply decide to bring actual attitudes in line with their ideal attitudes. Research shows that people do, in fact, act in ways that facilitate this alignment (DeMarree, et al., 2017; Vaughan-Johnston, Fabrigar, Xia, DeMarree, & Clark, 2023), but there are obstacles to this in the form of reality constraints and structural constraints (Wheeler & DeMarree, 2019). In brief, reality constraints concern limitations on one’s ability to completely shape their experiential reactions to objects. Although one can learn to like things one initially dislikes (e.g., exercise) and vice versa, not all experiences are amenable to easy manipulation, despite the use of epistemic and teleologic tactics such as reinterpretation or suppression (see Maio & Thomas, 2007). Sometimes one’s experienced responses to a stimulus (e.g., the taste of liver) are resistant to reformulation or change. Structural constraints concern the array of cognitive components (e.g., beliefs, identities, values, motivations, etc.) that are mentally associated with the ideal attitude in question. These can include not just one’s own cognitive components but also those of others (i.e., interpersonal congruence). Because ideal attitudes exist in a potentially vast network of associated components, not all of which may themselves be consistent, achieving perfect consonance between all components in the network may be impossible.

Distinction Between Actual-ideal Preference Discrepancies and Related Constructs

An individual's ideal preferences can be more normatively virtuous than one's actual preferences (e.g., wanting to like exercising more than one actually does), less normatively virtuous that one's actual preferences (e.g., wanting to like scotch more than one actually does), or unrelated to virtue altogether (e.g., wanting to like one's apartment more than one actually does). As these examples illustrate, the actual-ideal preference distinction differs from those of related constructs. For example, though research on actual-ideal preference discrepancies shares terminology with the ideal selves of regulatory focus theory (Higgins, 2011), actual-ideal preference discrepancies need have nothing to do with approach vs. avoidance orientations, goal pursuit, or the self-concept. Some actual-ideal preference discrepancies stem from strictly pragmatic concerns (e.g., wanting to like things simply because they are popular, or wanting to like things that one owns or uses, as in the case of the apartment example above). For similar reasons, they are distinct from want-should conflicts (Milkman et al., 2008). Actual-ideal preference discrepancies also need not involve any conflict between short-term and long-term desires, which makes them distinct from intertemporal tradeoffs (Frederick et al., 2003).

The reader may question why we did not also study ought attitudes, that is, the attitudes one feels one ought to hold. There are two reasons for this: First, ideal attitudes have been shown to be stronger predictors of behavior (DeMarree et al., 2017), perhaps because they reflect personal, rather than interpersonal standards for behavior. Second, showing effects of recommending content aligned with one's individually determined ideal attitudes (vs. one's socially determined ought attitudes) would illustrate the benefit of truly personalizing content to an individual's desired preferences, rather than to normative societal prescriptions.

Timing Analyses

We ran additional analyses to examine how long participants spent reading the recommended article. Because the reading time measure was skewed, we log-transformed this measure for the following analyses. However, the statistical conclusions remain unchanged when using the untransformed measure. We began by comparing reading time between the actual and ideal conditions. Because the personalized recommendations for each condition may have differed in article length and other ways, we used several different approaches to account for differences in articles. First, we ran a mixed-effects linear model in which we regressed log-transformed reading time on condition with a random intercept for the recommended article; there was no statistically significant difference between conditions ($b = 0.04$, 95% CI = [-0.05, 0.13], $t(2467) = 0.86$, $p = .393$). In addition, we ran a linear model in which we regressed log-transformed reading time on condition and word count, controlling for the interaction between the two; as before, there was no statistically significant effect of condition on reading time ($b = 0.11$, 95% CI = [-0.17, 0.39], $t(2500) = 0.79$, $p = .433$) and the interaction was not significant either ($b = -0.00$, 95% CI = [-0.00, 0.00], $t(2500) = -0.82$, $p = .412$).

In addition to comparing condition, we also examined the relationship between the predicted degree of discrepancy that participants felt toward the recommended article and log-transformed reading time. Because the machine learning models generated point predictions for how much each person actually and ideally wanted to read the recommended article, we could calculate the difference between these values to serve as an estimate of how much people experienced tension between their actual and ideal preferences for the recommended article. This allowed for a more granular approach than using condition alone. For example, even for participants in the same condition, some participants could have a bigger predicted discrepancy

than others for a particular article, and this presumably greater tension could perhaps affect reading time. Conducting similar analyses as before, we ran a mixed-effects linear model in which we regressed log-transformed reading time on predicted preference discrepancy with a random intercept for the recommended article; there was no statistically significant effect of predicted preference discrepancy on reading time (b = 0.00, 95% CI = [-0.00, 0.01], t(1779) = 0.59, p = .555). When we ran a linear model in which we regressed log-transformed reading time on predicted preference discrepancy and word count, controlling for the interaction between the two, there was no statistically significant effect of predicted preference discrepancy on reading time (b = 0.00, 95% CI = [-0.00, 0.01], t(2500) = 0.57, p = .566), and the interaction was not significant either (b = -0.00, 95% CI = [-0.00, 0.00], t(2500) = -0.60, p = .546).

In addition, we ran analyses to examine how long it took participants to decide whether to read the recommended article. We used log-transformed choice time to correct for skew in the following analyses; however, as before, the statistical conclusions remain unchanged when using the untransformed measure. We began by comparing choice time between the actual and ideal conditions. We used a mixed-effects linear model to regress log-transformed choice time on condition with a random intercept for the article and found no significant differences between conditions (b = 0.01, 95% CI = [-0.03, 0.05], t(2502) = 0.63, p = .529). In addition to comparing condition, we also examined the relationship between the predicted degree of discrepancy that participants felt toward the recommended article and log-transformed choice time. We ran a mixed-effects linear model in which we regressed log-transformed choice time on predicted preference discrepancy with a random intercept for the recommended article; there was no statistically significant impact of predicted preference discrepancy on choice time (b = -0.00, 95% CI = [-0.00, 0.00], t(2502) = -1.32, p = .188).

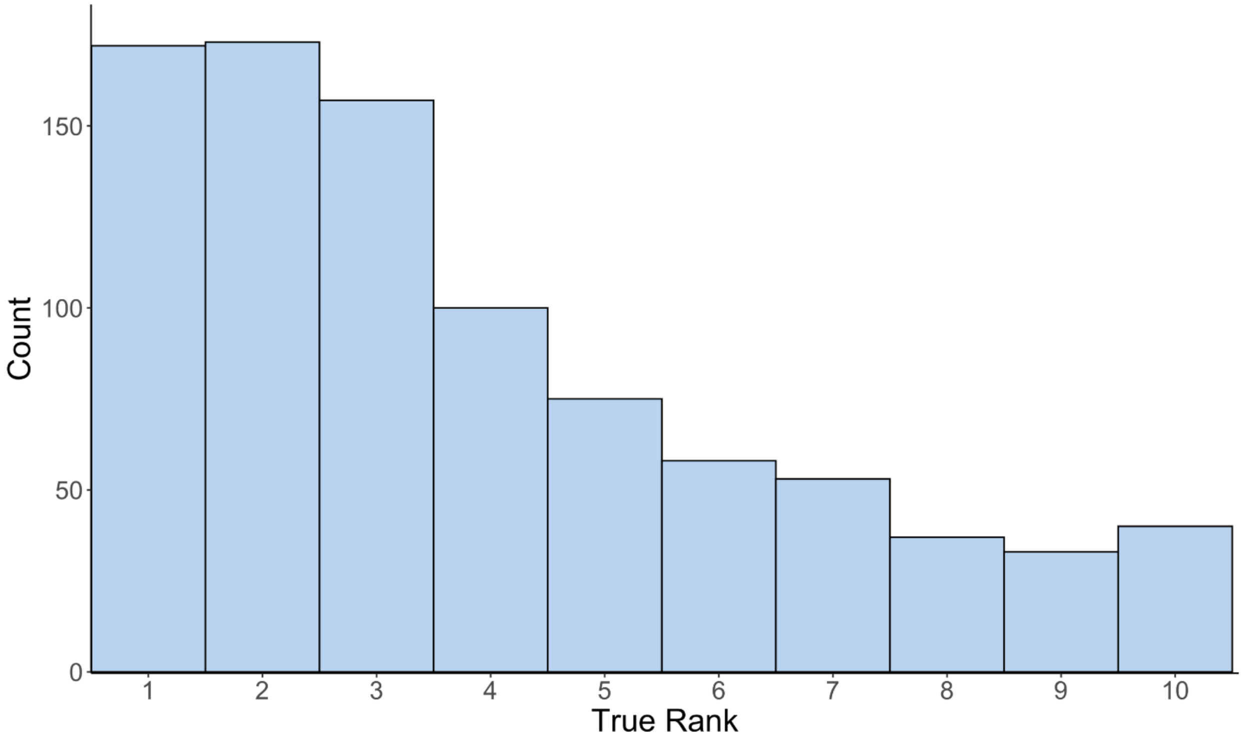

Fig. S1. True rank of article predicted to be highest (actual). This histogram depicts the true preference rank (out of 10) of the article that the algorithm predicted to be highest on actual preference. Predictions were generated using 10-fold cross validation.

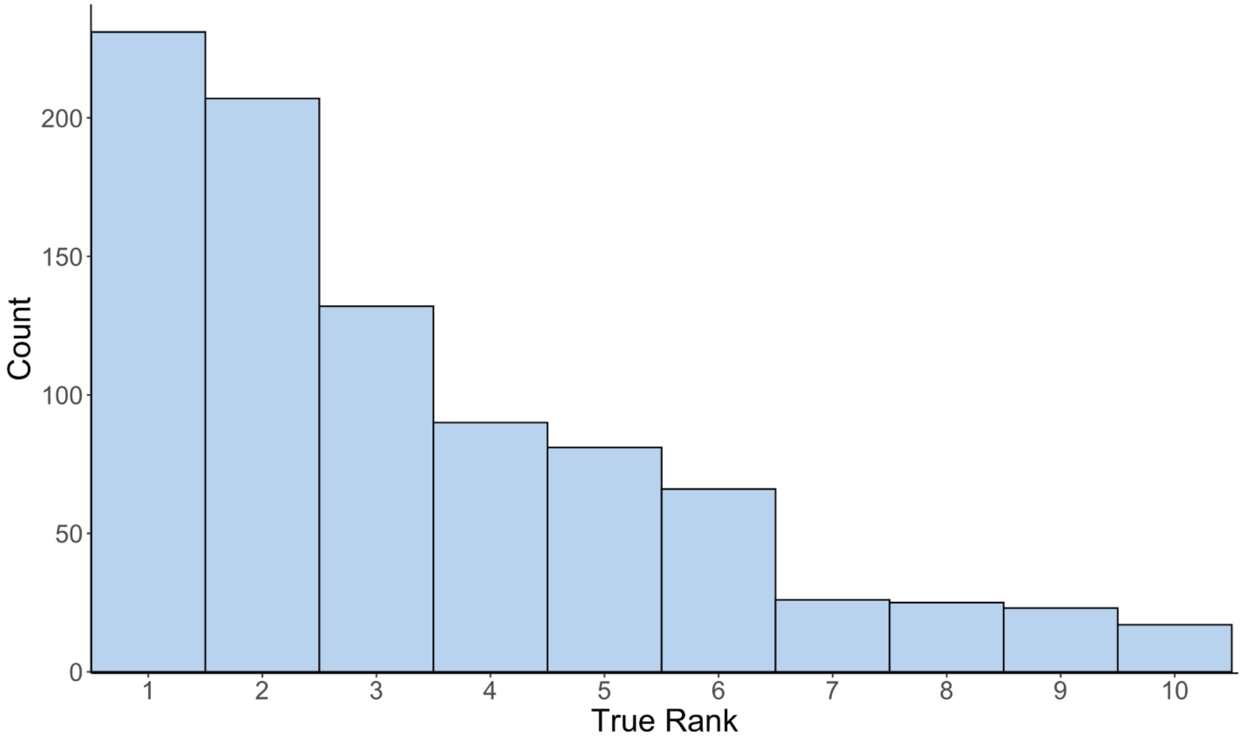

Fig. S2. True rank of article predicted to be highest (ideal). This histogram depicts the true preference rank (out of 10) of the article that the algorithm predicted to be highest on ideal preference. Predictions were generated using 10-fold cross validation.

Dependent Variable	Random				Actual				Ideal			
	N	*M*	*SD*	95% CI	*N*	*M*	*SD*	95% CI	*N*	*M*	*SD*	95% CI
Helpfulness of Recommendation	2,154	3.15	1.90	[3.07, 3.23]	2,171	4.38	1.87	[4.30, 4.46]	2,163	4.65	1.78	[4.58, 4.73]
Better Off Receiving Recommendation	2,154	2.85	1.82	[2.78, 2.93]	2,171	4.00	1.92	[3.92, 4.09]	2,163	4.41	1.78	[4.33, 4.48]
Company Has Best Interest at Heart	2,154	2.95	1.79	[2.87, 3.02]	2,171	3.91	1.82	[3.83, 3.98]	2,163	4.22	1.69	[4.14, 4.29]
Willingness to Pay for Service	2,154	1.40	2.94	[1.28, 1.53]	2,171	2.15	3.66	[2.00, 2.31]	2,163	2.27	3.63	[2.12, 2.43]
Chose to Read Recommended Article	2,154	0.35	0.48	[0.33, 0.37]	2,171	0.54	0.50	[0.52, 0.56]	2,163	0.48	0.50	[0.46, 0.50]
Liked Reading Article	2,154	3.24	1.91	[3.16, 3.32]	2,171	4.24	1.85	[4.16, 4.31]	2,163	4.03	1.88	[3.95, 4.11]
Enjoyed Reading Article	2,154	3.12	1.87	[3.05, 3.20]	2,171	4.11	1.85	[4.03, 4.19]	2,163	3.81	1.86	[3.73, 3.89]
Better Off After Reading Article	2,154	2.74	1.82	[2.66, 2.81]	2,171	3.74	1.93	[3.66, 3.82]	2,163	4.15	1.86	[4.07, 4.23]
Likelihood of Reading Similar Articles	2,154	3.16	1.99	[3.08, 3.25]	2,171	4.35	1.95	[4.27, 4.44]	2,163	4.30	1.96	[4.22, 4.38]
Time Well Spent	2,154	3.15	1.96	[3.07, 3.24]	2,171	4.22	1.93	[4.14, 4.30]	2,163	4.46	1.91	[4.38, 4.54]
Likelihood of Using Service Again	2,154	2.66	1.86	[2.59, 2.74]	2,171	3.62	1.96	[3.54, 3.70]	2,163	3.66	1.93	[3.58, 3.74]

Table S1. Summary statistics for all conditions and all participants. Data include participants who would have received the same recommendation in both the actual and ideal conditions. For the full text of dependent variables, see Table 1.

Dependent Variable	Actual				Ideal			
	b	95% CI	*t* / *z*	*p*	*b*	95% CI	*t* / *z*	*p*
Helpfulness of Recommendation	1.23	[1.12, 1.34]	21.80	<.001	1.50	[1.39, 1.61]	26.63	<.001
Better Off Receiving Recommendation	1.15	[1.04, 1.26]	20.55	<.001	1.55	[1.44, 1.66]	27.70	<.001
Company Has Best Interest at Heart	0.96	[0.85, 1.06]	17.83	<.001	1.27	[1.16, 1.37]	23.51	<.001
Willingness to Pay for Service	0.75	[0.55, 0.95]	7.20	<.001	0.87	[0.67, 1.08]	8.35	<.001
Chose to Read Recommended Article[a]	0.77	[0.65, 0.90]	12.41	<.001	0.53	[0.41, 0.66]	8.56	<.001
Liked Reading Article	1.00	[0.88, 1.11]	17.47	<.001	0.79	[0.68, 0.90]	13.83	<.001
Enjoyed Reading Article	0.99	[0.88, 1.10]	17.47	<.001	0.69	[0.58, 0.80]	12.13	<.001
Better Off After Reading Article	1.00	[0.89, 1.11]	17.64	<.001	1.41	[1.30, 1.52]	24.84	<.001
Likelihood of Reading Similar Articles	1.19	[1.08, 1.31]	19.92	<.001	1.14	[1.02, 1.26]	19.04	<.001
Time Well Spent	1.07	[0.95, 1.19]	18.19	<.001	1.30	[1.19, 1.42]	22.16	<.001
Likelihood of Using Service Again	0.95	[0.84, 1.07]	16.37	<.001	0.99	[0.88, 1.11]	17.02	<.001

Table S2. Regression results for all conditions and all participants. All dependent variables were predicted by condition with the random condition used as the baseline (n = 6,488).

[a] This dichotomous variable was analyzed using a binomial logistic regression model. All other variables were continuous and analyzed using linear regression models.

Dependent Variable	Actual				Ideal				Significance Tests			
	N	*M*	*SD*	95% CI	*N*	*M*	*SD*	95% CI	*df*	*t / z*	*p*	*d / OR*
Helpfulness of Recommendation	1,264	3.83	1.94	[3.72, 3.94]	1,240	4.32	1.85	[4.21, 4.42]	2500.03	6.37	<.001	0.25
Better Off Receiving Recommendation	1,264	3.37	1.93	[3.26, 3.48]	1,240	4.06	1.85	[3.95, 4.16]	2500.36	9.10	<.001	0.36
Company Has Best Interest at Heart	1,264	3.43	1.81	[3.33, 3.53]	1,240	4.00	1.76	[3.90, 4.10]	2500.76	8.04	<.001	0.32
Willingness to Pay for Service	1,264	1.84	3.47	[1.65, 2.03]	1,240	2.19	3.61	[1.99, 2.39]	2492.00	2.53	0.012	0.10
Chose to Read Recommended Article	1,264	0.52	0.50	[0.50, 0.55]	1,240	0.40	0.49	[0.37, 0.43]	2502.00	-6.23	<.001	0.60
Liked Reading Article	1,264	4.12	1.92	[4.02, 4.23]	1,240	3.77	1.91	[3.67, 3.88]	2501.55	-4.61	<.001	-0.18
Enjoyed Reading Article	1,264	4.02	1.93	[3.91, 4.12]	1,240	3.52	1.88	[3.41, 3.62]	2501.94	-6.54	<.001	-0.26
Better Off After Reading Article	1,264	3.20	1.90	[3.10, 3.31]	1,240	3.91	1.92	[3.80, 4.01]	2499.28	9.19	<.001	0.37
Likelihood of Reading Similar Articles	1,264	4.09	2.02	[3.97, 4.20]	1,240	4.01	2.02	[3.89, 4.12]	2500.01	-0.98	0.328	-0.04
Time Well Spent	1,264	3.87	1.99	[3.76, 3.98]	1,240	4.26	2.00	[4.15, 4.37]	2500.62	4.94	<.001	0.20
Likelihood of Using Service Again	1,264	3.30	1.97	[3.20, 3.41]	1,240	3.48	1.97	[3.37, 3.59]	2500.79	2.20	0.028	0.09

Table S3. Summary statistics and significance tests comparing the actual and ideal conditions for participants with a preference discrepancy. Data include only participants with a preference discrepancy (i.e., those who would have received different recommendations in the actual and ideal conditions). For continuous measures, two-tailed Welch Two Sample t-tests were used to evaluate significance, and Cohen's *d* was used to estimate effect size. Although we pre-registered one-tailed t-tests to increase our statistical power(Lakens, n.d.), this table reports results from the more conservative two-tailed t-tests. For the sole dichotomous measure, choosing to read the recommended article, a logistic regression was used to evaluate significance, and the odds ratio (*OR*) was used to estimate effect size. We note that unlike the other positive effect sizes displayed, the effect of the actual condition was stronger than the effect of the ideal condition for this dependent variable, as evidenced by the odds ratio, which always takes on positive values, being less than 1.

Dependent Variable	Model 1 *b*	Model 1 95% CI	Model 1 *t* / *z*	Model 1 *p*	Model 2 *b*	Model 2 95% CI	Model 2 *t* / *z*	Model 2 *p*	Model 3 *b*	Model 3 95% CI	Model 3 *t* / *z*	Model 3 *p*
Helpfulness of Recommendation	0.48	[0.33, 0.63]	6.37	<.001	0.47	[0.32, 0.62]	6.24	<.001	0.48	[0.34, 0.62]	6.77	<.001
Better Off Receiving Recommendation	0.69	[0.54, 0.83]	9.09	<.001	0.68	[0.53, 0.83]	9.09	<.001	0.69	[0.56, 0.83]	9.83	<.001
Company Has Best Interest at Heart	0.57	[0.43, 0.71]	8.04	<.001	0.57	[0.43, 0.71]	7.96	<.001	0.58	[0.45, 0.71]	8.55	<.001
Willingness to Pay for Service	0.36	[0.08, 0.63]	2.53	0.011	0.34	[0.07, 0.61]	2.43	0.015	0.36	[0.10, 0.61]	2.73	0.006
Chose to Read Recommended Article[a]	-0.50	[-0.66, -0.35]	-6.23	<.001	-0.53	[-0.69, -0.37]	-6.52	<.001	-0.54	[-0.71, -0.38]	-6.48	<.001
Liked Reading Article	-0.35	[-0.50, -0.20]	-4.61	<.001	-0.37	[-0.52, -0.22]	-4.82	<.001	-0.36	[-0.50, -0.22]	-5.01	<.001
Enjoyed Reading Article	-0.50	[-0.65, -0.35]	-6.54	<.001	-0.51	[-0.66, -0.37]	-6.80	<.001	-0.50	[-0.65, -0.36]	-7.04	<.001
Better Off After Reading Article	0.70	[0.55, 0.85]	9.19	<.001	0.69	[0.55, 0.84]	9.12	<.001	0.71	[0.56, 0.85]	9.77	<.001
Likelihood of Reading Similar Articles	-0.08	[-0.24, 0.08]	-0.98	0.328	-0.09	[-0.25, 0.07]	-1.15	0.249	-0.09	[-0.24, 0.06]	-1.18	0.237
Time Well Spent	0.39	[0.24, 0.55]	4.94	<.001	0.38	[0.23, 0.54]	4.82	<.001	0.40	[0.25, 0.54]	5.25	<.001
Likelihood of Using Service Again	0.17	[0.02, 0.33]	2.20	0.028	0.16	[0.01, 0.32]	2.08	0.038	0.17	[0.03, 0.32]	2.36	0.018

Table S4. Regression results comparing the actual and ideal conditions for participants with a preference discrepancy with controls. Unstandardized coefficients and corresponding statistics are reported for the effect of the ideal (vs. actual) condition on all dependent variables (n = 2,504). To test for robustness, multiple nested models were used. Model 1 contained only the condition as the independent variable. Model 2 contained the condition as well as demographic controls (gender, age, income, political preferences, and religious views). Model 3 contained the condition, demographic controls, news preferences, and news consumption frequency. All patterns remained consistent across all models.

[a] This dichotomous variable was analyzed using a binomial logistic regression model. All other variables were continuous and analyzed using linear regression models.

	Actual					Ideal					Significance Tests			
Dependent Variable	*N*	*M*	*SD*	*SE*	95% CI	*N*	*M*	*SD*	*SE*	95% CI	*df*	*t*	*p*	*d*
Liked Reading Article	662	4.92	1.66	0.06	[4.79, 5.05]	495	4.64	1.74	0.08	[4.49, 4.80]	1035.93	-2.73	0.007	-0.16
Enjoyed Reading Article	662	4.77	1.74	0.07	[4.63, 4.90]	495	4.36	1.78	0.08	[4.21, 4.52]	1050.41	-3.85	<.001	-0.23
Better Off After Reading Article	662	3.75	1.92	0.07	[3.60, 3.89]	495	4.67	1.71	0.08	[4.52, 4.83]	1119.88	8.63	<.001	0.50
Likelihood of Reading Similar Articles	662	4.99	1.75	0.07	[4.86, 5.12]	495	4.98	1.76	0.08	[4.82, 5.13]	1059.63	-0.15	0.884	-0.01
Time Well Spent	662	4.55	1.87	0.07	[4.40, 4.69]	495	5.01	1.74	0.08	[4.86, 5.17]	1102.64	4.38	<.001	0.26
Likelihood of Using Service Again	662	4.02	1.91	0.07	[3.88, 4.17]	495	4.32	1.89	0.08	[4.15, 4.49]	1070.47	2.65	0.008	0.16

Table S5. Summary statistics and significance tests for post-reading reactions of participants who chose to read the recommended article. Data include post-reading measures for only participants with a preference discrepancy who *chose* to read the recommended article. For all measures, t-tests were used to evaluate significance, and Cohen's *d* was used to estimate effect size.

Dependent Variable	Actual					Ideal					Significance Tests			
	N	*M*	*SD*	*SE*	95% CI	*N*	*M*	*SD*	*SE*	95% CI	*df*	*t*	*p*	*d*
Liked Reading Article	602	3.25	1.79	0.07	[3.10, 3.39]	745	3.19	1.79	0.07	[3.06, 3.32]	1283.92	-0.57	0.572	-0.03
Enjoyed Reading Article	602	3.19	1.79	0.07	[3.05, 3.34]	745	2.96	1.74	0.06	[2.83, 3.08]	1269.59	-2.44	0.015	-0.13
Better Off After Reading Article	602	2.61	1.68	0.07	[2.47, 2.74]	745	3.40	1.89	0.07	[3.26, 3.53]	1332.46	8.11	<.001	0.44
Likelihood of Reading Similar Articles	602	3.09	1.82	0.07	[2.94, 3.23]	745	3.36	1.93	0.07	[3.22, 3.50]	1310.82	2.68	0.008	0.15
Time Well Spent	602	3.12	1.84	0.07	[2.97, 3.26]	745	3.76	2.00	0.07	[3.61, 3.90]	1322.83	6.11	<.001	0.33
Likelihood of Using Service Again	602	2.51	1.71	0.07	[2.38, 2.65]	745	2.92	1.82	0.07	[2.79, 3.05]	1315.05	4.16	<.001	0.23

Table S6. Summary statistics and significance tests for post-reading reactions of participants who chose not to read the recommended article. Data include post-reading measures for only participants with a preference discrepancy who *chose not* to read the recommended article. For all measures, t-tests were used to evaluate significance, and Cohen's *d* was used to estimate effect size.

	Interaction Term			
Dependent Variable	*b*	95% CI	*t*	*p*
Liked Reading Article	-0.22	[-0.50, 0.06]	-1.57	0.117
Enjoyed Reading Article	-0.17	[-0.44, 0.11]	-1.17	0.242
Better Off After Reading Article	0.14	[-0.15, 0.43]	0.94	0.348
Likelihood of Reading Similar Articles	-0.29	[-0.58, 0.00]	-1.96	0.050
Time Well Spent	-0.17	[-0.47, 0.12]	-1.14	0.254
Likelihood of Using Service Again	-0.10	[-0.39, 0.19]	-0.70	0.483

Table S7. Interaction of condition and choice to read recommended article on post-reading dependent variables. Data only includes participants with a preference discrepancy.

References

DeMarree, K. G., Clark, C. J., Wheeler, S. C., Briñol, P., & Petty, R. E. (2017). On the pursuit of desired attitudes: Wanting a different attitude affects information processing and behavior. Journal of Experimental Social Psychology, 70, 129–142. https://doi.org/10.1016/j.jesp.2017.01.003

Ferguson, M. J. (2007). On the automatic evaluation of end-states. Journal of Personality and Social Psychology, 92, 596–611. https://doi.org/10.1037/0022-3514.92.4.596

Ferguson, M. J. (2008). On becoming ready to pursue a goal you don't know you have: Effects of nonconscious goals on evaluative readiness. Journal of Personality and Social Psychology, 95, 1268–1294. https://doi.org/10.1037/a0013263

Festinger, L. (1957). A theory of cognitive dissonance. Stanford University Press.

Frederick, S., Loewenstein, G., & O'Donoghue, T. (2003). Time discounting and time preference: A critical review. *Time and Decision: Economic and Psychological Perspectives on Intertemporal Choice*, *XL*(June), 13–86. https://doi.org/10.1257/002205102320161311

Fitzsimons, G. M., & Shah, J. Y. (2008). How goal instrumentality shapes relationship evaluations. Journal of Personality and Social Psychology, 95, 319–337. https://doi.org/10.1037/0022-3514.95.2.319

Heider, F. (1958). The psychology of interpersonal relations. Wiley.

Higgins, E. T. (2011). Regulatory focus theory. In *Handbook of Theories of Social Psychology*. SAGE Publications.

Jordan, N. (1953). Behavioral Forces That are a Function of Attitudes and of Cognitive Organization. Human Relations, 6(3), 273–287. https://doi.org/10.1177/001872675300600304

Lakens, D. (n.d.). *Will knowledge about more efficient study designs increase the willingness to pre-register?* https://doi.org/10.31222/osf.io/svzyc

Maio, G. R., & Thomas, G. (2007). The epistemic-teleologic model of deliberate self-persuasion. Personality and Social Psychology Review, 11(1), 1–22.

Milkman, K. L., Rogers, T., & Bazerman, M. H. (2008). Harnessing our inner angels and demons: What we have learned about want/should conflicts and how that knowledge can help us reduce short-sighted decision making. *Perspectives on Psychological Science*, *3*(4), 324–338. https://doi.org/10.1111/j.1745-6924.2008.00083.x

Priester, J. R., & Petty, R. E. (2001). Extending the bases of subjective attitudinal ambivalence: Interpersonal and intrapersonal antecedents of evaluative tension. Journal of Personality & Social Psychology, 80(1), 19–34.

Vaughan-Johnston, T. I., Fabrigar, L. R., Xia, J., DeMarree, K. G., & Clark, J. K. (2023). Desired attitudes guide actual attitude change. Journal of Experimental Social Psychology, 105, 104437. https://doi.org/10.1016/j.jesp.2022.104437

Wheeler, S. C., & Bechler, C. J. (2020). Objects and Self-identity. Current Opinion in Psychology, 39, 6–11. https://doi.org/10.1016/j.copsyc.2020.07.013

Wheeler, S. C., & DeMarree, K. G. (2019). Chapter 23: Prevalence, antecedents and consequences of actual_desired attitude discrepancies in: Handbook of Research on Identity Theory in Marketing. In Handbook of Research on Identity Theory in Marketing (pp. 346–359). Elgar.
